# The impact of dental metal restorations on the oral oxidative stress level

**DOI:** 10.4317/jced.60175

**Published:** 2023-03-01

**Authors:** Zlatina Tomova, Desislav Tomov, Angelina Vlahova

**Affiliations:** 1 DMD, PhD, chief assistant professor, Department of Prosthetic dental medicine, Faculty of dental medicine, Medical University of Plovdiv, Bulgaria; 2 MD, Department of Bioorganic chemistry, Faculty of Pharmacy, Medical University of Plovdiv; Plovdiv, Bulgaria; 3 DMD, PhD, professor, head of the Department of Prosthetic Dental medicine, Faculty of Dental Medicine, Medical University Plovdiv, Bulgaria

## Abstract

**Background:**

Dental materials may influence the equilibrium between production and destruction of free radicals, thus creating conditions for developing of local or general oxidative stress. Metal ions, emitted from base dental alloys, may cause changes in cell structures and functions. Isoprostane concentration may indicate possible cell damage, caused by free radicals, and can be used for evaluation of the oxidative stress level. The aim of this study was to compare the level of 8-isoPGF2-alpha in saliva in patients with and without metal dental restorations.

**Material and Methods:**

35 patients were divided in two groups according to the presence or absence of metal dental objects. Non-stimulated and stimulated saliva samples were collected. The concentration of 8-isoPGF2-alpha was measured by liquid chromatography tandem mass spectrometry. For statistical analysis non-parametric Mann-Whitney test, Kruskal Wallis test, and Wilcoxon signed-rank test were applied.

**Results:**

There was a significant difference in the concentration of 8-isoPGF2-alpha between the samples of non-stimulated and stimulated saliva. The concentration of 8-isoPGF2-alpha in non-stimulated saliva in patients with metal dental restorations was significantly higher than the one in the group of patients without metal objects.

**Conclusions:**

The presence of metal dental restorations increases the concentration of 8-isoPGF2-alpha in non-stimulated saliva.

** Key words:**Saliva, dental metal restorations, oxidative stress.

## Introduction

Oral cavity is the place, in which different in nature foreign agents enter the body. These substances may influence the equilibrium between production and destruction of ROS (reactive oxygen species) and RNS (reactive nitrogen species), thus creating conditions for developing state of local or general oxidative stress. The connection between oxidative stress and pathological alterations in the body is bidirectional. Oxidative stress may be induced by some general diseases like diabetes, rheumatoid arthritis, chronic renal failure, Crohn’s disease etc, (1). Some oral pathologies, like periodontal inflammatory diseases, may cause local oxidative stress, while others like leukoplakia, oral cancer, and recurrent aphthous stomatitis may increase the level of ROS in the blood plasma and the whole human body (2). On the other side, oxidative stress contributes to various pathophysiological changes and is involved in multiple stages of carcinogenesis. In the oral cavity oxidative stress leads to progression of chronic inflammation, degradation of the extracellular matrix of the periodontium, and resorption of the bone (3).

There are numerous factors that may induce increased production of free radicals in the oral cavity (4). Gingival and periodontal inflammatory diseases appear as a response to pathogenic oral microorganisms. The production of ROS in the inflammatory response plays a key role in the neutralization and elimination of bacteria. The increased amount of ROS, however, affects not only the pathological agents but also the tissues of the host (5). High-fat and high-protein diet and the way food is prepared may also be a source of ROS entering the body, although it is worth mentioning that antioxidants are taken with food. Containing many pro-oxidative and carcinogenic substances, cigarette smoke is one of the most powerful oxidative stress inducers in the oral cavity. Alcohol consumption also affects the redox balance in the oral cavity (4). Dental materials, like resin composites, different types of cements, root canal fillings and dental alloys, may disturb the local balance between formation and neutralization of free radicals (6). Laser treatment of the soft and hard dental tissues is an important source of free radicals in the oral cavity. Laser photodynamic therapy is based on the increased production of ROS and their potential bactericidal action (7). Recent study shows that bisphosphonate therapy in treatment of osteoporosis causes oxidative stress in oral fibroblasts leading to possible osteonecrosis of the jaws (8).

Foreign to the body materials with different chemical, mechanical and biological properties are used for dental treatment. They must provide longevity, esthetics, and safe use. Base dental alloys (nickel-chromium and cobalt-chromium) are used for production of removable and fixed prosthetic restorations. They are preferred because of the excellent mechanical properties that can withstand the forces during the mastication process, and because of the comparably low price. With the development and popularization of CAD/CAM technologies the production of metal-free restorations increases. However, metal ceramic prosthetic devices are still more common probably because of financial reasons. The most important feature of the dental alloy for its biocompatibility is the tendency to corrosion. Metal ions, emitted due to corrosion process, come in direct contact with the surrounding soft tissues and may enter the body through the gastrointestinal tract. They may cause allergic reactions, systemic and local toxicity, cancerogenic alterations, changes in cell structures and functions. It is suggested that even if the concentration of metal ions has no direct cytotoxic effect, it may have influence at molecular level and may contribute to alteration in the immune system response in patients with implants made of cobalt-chromium alloy (9). Nickel and cobalt ions may react with hydrogen peroxide and via the Fenton reaction may generate hydroxyl radicals. Chromium and cobalt ions may undergo redox cycling, thus directly producing free radicals (10).

Saliva is a complex fluid, and although it consists of more than 95% water, it also contains many organic and inorganic components. The average flow rate of non-stimulated saliva is about 0.3 mL/min with great individual variability and circadian rhythm. There is a significant difference in the flow rate and the composition of stimulated and non-stimulated saliva. Saliva provides non-invasive, painless, cost-effective, and fast sample collection and may be used as an alternative testing medium of blood and urine. Although it is not widely utilized biological fluid for metabolite analysis, the number of studies using it increases. Methods for analysing levels of different substances – cortisol, melatonin, creatinine, SARS-CoV-2 antibodies, etc., are already developed (11).

Formation of 8-isoPGF2-alpha is a result of interaction of free radicals with lipids, present in cell membranes. Isoprostane concentration may indicate possible cell damage. There are two big advantages of using isoprostanes as oxidative stress marker – presence in all fluids in the body and low reactivity. Furthermore, their local concentration may be used for evaluation and observation of the specific area of the body (12).

The aim of this study was to compare the salivary level of 8-isoPGF2-alpha as marker of oxidative stress in patients with and without metal restorations in the oral cavity.

Materials and methods: 35 participants were divided in two groups according to the presence or absence of present metal restorations in the oral cavity – 17 of the volunteers had no metal objects in the mouth and 18 of them had metal dental restorations. All the patients included in the study have signed informed consent. All procedures performed in the studies were in accordance with the standards of the Institutional Committee of Scientific Ethics of Medical University of Plovdiv, Bulgaria (Decision № С – 03-2/10.04.2020) and with the Association Declaration of Helsinki from 1964. The participants had to meet the following criteria: non-smokers at the age between 18 and 65, without acute or chronic diseases. None of the patients reported symptoms of gastric disorders and no signs of acidic erosion on the hard dental tissues were found.

Saliva samples were gathered in the dental office by spitting in low density polyethylene containers in the interval between 9.00 A.M. and 12.00 A.M. without exposure to any visual, taste or aromatic stimuli. Patients were instructed not to take any food or drinks except water before the dental visit. Non-stimulated and stimulated saliva samples were taken from the patients before any dental procedures were conducted. After rinsing the mouth with distilled water, the patients spat the saliva gathered at the bottom of the oral cavity for 15-20 minutes. After gathering 15 ml of non-stimulated saliva, 5 ml were placed in a centrifugal tube for detection of isoprostane 8-isoPGF2-alpha. Stimulated saliva samples were taken after placing 2% citric acid over the tongue (100 µL each 30 seconds) for 5 minutes. The samples were immediately frozen at -20oC and later transferred to the Research Institute at Medical University of Plovdiv for storage at -70oC. For evaluation of oxidative stress level in the oral cavity concentration of 8-isoPGF2-alpha was measured by liquid chromatography tandem mass spectrometry (LC-MS/MS). An LC-MS/MS method was developed and validated for detection of 8-isoPGF2-alpha in saliva (13).

For statistical processing SPSS statistical package, version 19.0 was used. Non-parametric Mann-Whitney test, Kruskal Wallis Test, and Wilcoxon signed-rank test were applied. Level of significance was *p*<0.05.

## Results

The descriptive analysis of concentration of 8-isoPGF2-alpha in the studied groups is given at Table 1.

**Table 1:**
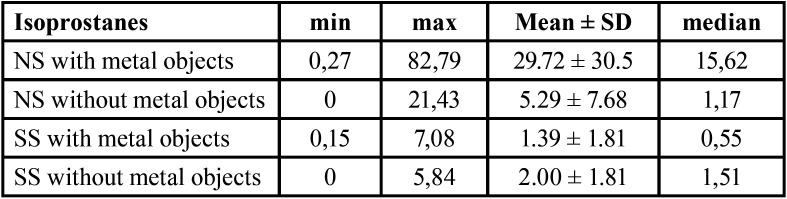
Concentration of 8-isoPGF2-alpha in non-stimulated (NS) and stimulated (SS) saliva in patients with and without metal dental restorations (ng/L).

Results showed that there was a signiFigant difference in the concentration of 8-isoPGF2-alpha in the samples of non-stimulated and stimulated saliva – for the group withiout metal object *p*=0.04 and for the group with metal restorations – *p*=0.001. Isoprostane concentration was higher in non-stimulated saliva.

**Figure 1:**
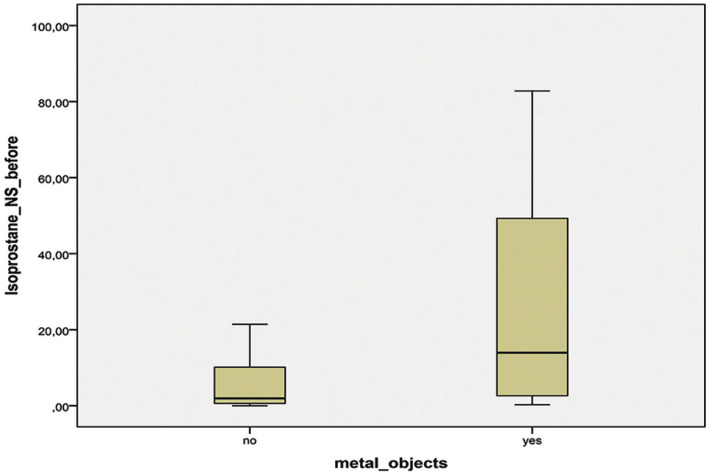
Concentration of 8-isoPGF2-alpha in non-stimulated saliva (NS) in the groups with (YES) and without (NO) metal objects.

**Figure 2:**
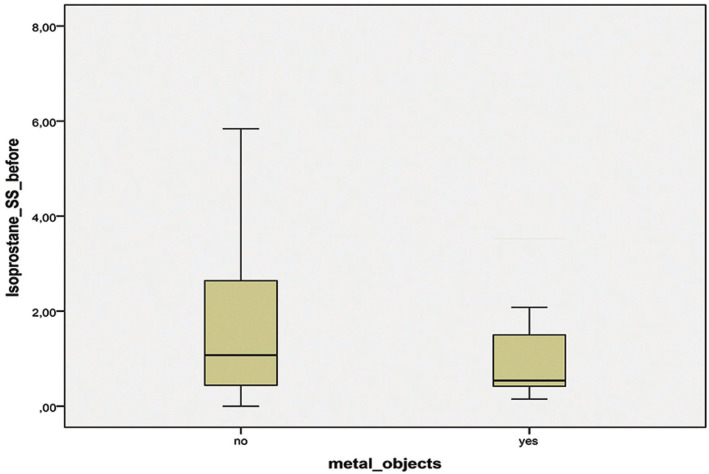
Concentration of 8-isoPGF2-alpha in stimulated saliva (SS) in the groups with (YES) and without (NO) metal objects.

The concentration of 8-isoPGF2-alpha in non-stimulated saliva in patients with metal dental restorations was significantly higher than the one in the group of patients without metal objects in the oral cavity – *p*=0.009 (Fig. 1). Comparing the stimulated saliva samples no significant differences were found in patients with and without metal restorations – *p*=0.294 (Fig. 2).

## Discussion

There are studies confirming significant daily variations in the concentration of some salivary oxidative stress markers (14). This was the reason all saliva samples for our research were gathered in the interval between 9.00 AM and 12.00 AM.

The results of our study suggest that the presence of metal restorations significantly increases the risk of developing oxidative stress in the oral cavity. According to the data gathered from the patients, all the metal prosthetic restorations had been in the mouth for more than 1 year, which means that the passivation process of the metal surfaces must have limited the corrosion process and the metal ion emission (15). Despite that fact, the results lead us to the conclusion that even after passivation the metal objects still influence the oxidative stress level in the oral cavity, i.e., the area which is in direct contact with the metal parts. The results of our study correspond to the findings of Kovač *et al*., 2020, according to which CoCr alloys may induce increase in oxidative stress level (16). Our study did not confirm the conclusions of McGinley *et al*., 2013, which found that dental CoCr alloys did not elicit adverse oxidative stress (17). It may be suggested that not only the presence, but also the composition of the alloy define the effect of the metal restoration placed in the mouth.

Non-stimulated saliva is a liquid that contains not only the saliva secreted from the salivary acini, but also products from the gingival fluid and from the biofilm covering all the surfaces in the oral cavity. Although stimulated saliva cannot be considered as analogue of blood plasma, it is clear from local affecting factors and may be used for analysis of the redox status of distant from the mouth areas. Zugla *et al*., 2019, found a correlation between levels of oxidative stress markers in saliva and plasma and concluded that saliva may be used as a medium for assessing the level of oxidative stress in the human body (18). From the insignificant difference in the isoprostane concentration in stimulated saliva samples between the groups with and without metal restorations it may be suggested that the presence of metal restorations did not affect distant areas of the body, which were not in a direct contact with them.

The limitation of this study was the small size of patients in the studied groups. The types of the alloys used for production of the metal prosthetic restorations of the patients could not be defined. Further investigations are needed to confirm the results.

## Conclusions

Within the limitations of this study, it can be concluded that the presence of metal dental restorations increases the concentration of 8-isoPGF2-alpha in non-stimulated saliva and hides a risk of developing of oxidative stress in the oral cavity.
